# Cognitive Therapy for Social Anxiety Disorder in Adolescents: A Development Case Series

**DOI:** 10.1017/S1352465815000715

**Published:** 2015-12-07

**Authors:** Eleanor Leigh, David M. Clark

**Affiliations:** 1 South London and Maudsley NHS Foundation Trust, and Institute of Psychiatry, Psychology and Neuroscience, Kings College London, UK; 2 University of Oxford, and NIHR Oxford Cognitive Health Clinical Research Facility, Oxford, UK

**Keywords:** Cognitive therapy, social anxiety, adolescent, young people

## Abstract

**Background:** Social anxiety disorder is common and typically starts in childhood or adolescence. Cognitive Therapy for Social Anxiety Disorder (CT-SAD) in adults is a well-established treatment that shows strong evidence of differential effectiveness when compared to other active treatments. In contrast, CBT approaches to social anxiety in young people have yet to demonstrate differential effectiveness and there is some evidence that young people with social anxiety disorder respond less well than those with other anxiety disorders. **Aims:** To adapt CT-SAD for use with adolescents and conduct a pilot case series. **Method:** Five adolescents, aged 11–17 years, with a primary DSM-5 diagnosis of social anxiety disorder received a course of CT-SAD adapted for adolescents. Standardized clinical interview and questionnaire assessments were conducted at pre and posttreatment, and 2 to 3-month follow-up. **Results:** All five participants reported severe social anxiety at baseline and achieved remission by the end of treatment. Significant improvements were also observed in general anxiety, depression, concentration in the classroom, and putative process measures (social anxiety related thoughts, beliefs and safety behaviours). **Conclusions**: An adapted form of CT-SAD shows promise as a treatment for adolescents.

## Introduction

Social anxiety is one of the most common and disabling anxiety disorders (Kessler et al., 2005) with a particularly low natural recovery rate (Bruce et al., 2005). The condition almost always starts in childhood or adolescence (Kessler et al., 2005) and can have profound effects on development that are hard to overcome later in life. A crowded classroom can make socially anxious youths self-conscious, making it difficult for them to follow what the teacher says, and consequently undermine exam performance. The development of social and romantic relationships is also impeded. Clearly, there is a major need for effective treatments that can be delivered in adolescence.

In adults, individual cognitive therapy for social anxiety disorder (CT-SAD) has a strong evidence base and has been shown to be superior to several other active treatments, including exposure therapy, group CBT, interpersonal psychotherapy, psychodynamic psychotherapy, selective serotonin re-uptake inhibitors, and medication based treatment as usual (Mayo-Wilson et al., 2014). The evidence base for CBT with young people is less strong (NICE, 2013). Generic CBT interventions for anxiety in young people appear to be less effective with social anxiety than other anxiety disorders (Ginsburg et al., 2011; Hudson et al., 2015). CBT programmes specifically developed for social anxiety in youth show a range of positive responses and may have a greater effect on social anxiety than generic CBT for young people. However, this has not yet been demonstrated. In addition, a recent meta-analysis (NICE, 2013) failed to establish that either generic or social anxiety specific CBT for youth is superior to other interventions, including psychological or pill placebos. In this uncertain situation, it is notable that the treatments that are most effective with adults have not, as yet, been trialled with adolescents. For this reason, we decided to conduct a development case series in which we would experiment with delivering individual CT-SAD based on the Clark and Wells (1995) model to a group of adolescents with severe social anxiety disorder. Our aim was twofold: 1) to provide a preliminary estimate of the potential utility of CT-SAD with adolescents; 2) to learn lessons about how its delivery might need to be modified for this population. We were encouraged to take this approach by studies that have shown that the psychological processes targeted by CT-SAD for adults are evident in adolescents. These include: similar negative cognitions (Cartwright-Hatton, Tschernitz and Gomersall, 2005), self-focused attention (Blote, Miers, Heyne, Clark and Westenberg, 2014), negative self-images (Schreiber and Steil, 2013), safety behaviours (Hodson, McManus, Clark and Doll, 2008), and postevent processing (Hodson et al., 2008). Furthermore, a version of CT-SAD that included some, but not all of the adult procedures, outperformed group CBT for young people and an attention control in a recent RCT with adolescents (Ingul, Aune and Nordahl, 2014).

## Method

### Participants

Participants were five young people aged 11 to 17 years who met DSM-5 (American Psychiatric Association, 2013) criteria for social anxiety disorder. All were referred to Child and Adolescent Mental Health Services for treatment. Table 1 shows patient characteristics. The Anxiety Disorders Interview Schedule for Children and Parents (ADIS-C/P; Silverman and Albano, 1996) was used as standard to diagnose social anxiety disorder and comorbid conditions. As is standard, a Clinician Severity Rating (CSR; 0 [absent or none] to 8 [very severely disturbing/disabling]) was assigned. A CSR of four or more indicates positive diagnosis. CSR scores at assessment are shown in Table 2. EL, who had extensive prior training with the ADIS C/P, conducted the interviews. Four of the five had previously received a course of the broad-based CBT that is commonly offered in children's services; in two cases (Patient 1 and 4) this specifically targeted social anxiety. Patient 4 had been on a stable dose of fluoxetine (20mg od) for 3 months prior to treatment. Patient 2 was not attending school and Patient 3 was an inconsistent school attender. Box 1 provides clinical vignettes.

**Table 1.** Participant details

P	Gender	Age (y)	Cognitions	Safety behaviours	Early events	Images
1	F	15	“People will laugh at me” “I am boring” “People will think I am stupid”	Talk less; avoid eye contact; try to give short answers; rush what I'm saying; talk quietly; monitor what I'm saying; plan answers; avoid asking questions; stay on the edge of groups; avoid participating	Standing in front of the class, unable to complete an oral presentation aged 11y	Separate from everyone, in the corner of the room, like a “stone”
2	F	11	*“*I am vulnerable” “I am weird” “I am inferior” “People will reject me”	Always agree with others; allow others to speak for me; avoid participating in class; follow dominant peer group	Losing control of bowels whilst seated in class unclear how to manage it aged 7y	Looking lost, someone who “doesn't have a clue”
3	F	16	“I am not interesting” “I am boring” “I will mash up my words” “I will sweat” “I will make people feel awkward”	Rehearse sentences in my mind; check how I'm coming across; talk less; plan topics in advance; try not to attract attention; avoid asking questions; try to picture how I appear to others; censor what I'm going to say; allow interruptions; check not sweating; keep arms down	At a birthday party getting the dress code wrong aged 6y	Someone that “just doesn't fit in”
4	M	17	“I am weird” “I am vulnerable” “People will stare at me” “I will mess up my words”	Avoid all eye contact; hide face in coat; talk less; avoid asking questions; sit away from other people; keep a tight control of my behaviour; plan what I am going to say; monitor what I'm saying	Chronic and severe bullying in two schools, having possessions stolen and being slapped in the face in front of peers, unable to retaliate aged 11y	A vulnerable, cowering helpless figure
5	F	15	“I am going to embarrass myself” “I will be rejected” “I won't have anything to say” “I won't be able to speak” “They'll think I'm immature and silly”	Talk less; ask lots of questions; plan topics in advance; monitor what I am saying; let others guide the conversation; agree with other people; pretend to share interests	Cried in front of class when teacher insulted favourite band, and the class laughed aged 11y. I thought they were laughing at me.	An “uncool, naïve, try-hard”

**Table 2.** Outcomes and process measures

		Patient					
		1	2	3	4	5	Mean (*SD*)
Clinical outcomes – social anxiety measures:							
LSAS							
	Pre	111	92	110	118	88	103.8 (13.1)
	Post	18	30	10	32	18	21.6 (9.2)
	Follow-up	0	7	9	29	15	12.0 (10.9)
SPWSS							
	Pre	5.2	5.3	5.2	5.5	6.5	5.5 (0.6)
	Post	0.7	0.3	0.7	2.0	2.2	1.2 (0.9)
	Follow-up	0.3	0.3	0	1.3	2.0	0.8 (0.8)
RCADS-P social anxiety							
	Pre	22	19	24	12	16	18.6 (4.8)
	Post	13	15	17	10	14	13.8 (2.6)
	Follow-up	4	8	13	8	9	8.4 (3.2)
ADIS-C/P social anxiety CSR ratings							
	Pre	7	6	6	7	6	6.4 (0.5)
	Post	0	1	2	3	2	1.6 (1.1)
	Follow-up	-	-	-	-	-	-
Social anxiety process measures:							
SCQ-F							
	Pre	3.5	4.1	3.0	2.9	4.2	3.5 (0.6)
	Post	1.3	1.9	1.2	1.0	1.3	1.3 (0.3)
	Follow-up	1.0	1.1	1.1	1.0	1.2	1.1(0.1)
SCQ-B							
	Pre	62.3	76.4	49.5	49.5	80.2	63.6 (14.5)
	Post	5.5	14.1	3.2	0.0	4.5	5.5 (5.3)
	Follow-up	4.1	0.5	2.3	0.0	2.0	1.8 (1.6)
SAQ							
	Pre	6.1	6.4	4.6	5.5	5.1	5.5 (0.7)
	Post	2.8	4.3	2.7	2.7	3.7	3.2 (0.7)
	Follow-up	1.5	1.6	3.3	2.1	3.4	2.4 (0.9)
SBQ							
	Pre	1.7	1.1	1.3	1.6	1.8	1.5 (0.3)
	Post	0.3	0.9	0.3	0.6	0.5	0.5 (0.3)
	Follow-up	0.1	0.0	0.3	0.6	0.3	0.3 (0.2)
Concentration:							
	Pre	22	40	37	40	15	42.8 (33.6)
	Post	79	79	80	100	50	77.6 (17.8)
	Follow-up	90	85	90	100	60	85.0 (15.0)
Clinical outcomes – general mood:							
GAD-7							
	Pre	8	10	9	11	10	9.6 (1.1)
	Post	1	0	6	1	11	3.8 (4.7)
	Follow-up	1	2	2	1	8	2.8 (2.9)
PHQ-9							
	Pre	10	5	9	14	13	10.2 (3.6)
	Post	3	2	12	4	18	7.8 (6.9)
	Follow-up	2	0	1	2	11	3.2 (4.4)

*Notes:* LSAS = Liebowitz Social Anxiety Scale (Baker et al., 2002); SPWSS = Social Phobia Weekly Summary Scale (Clarket al., 2003); RCADS = Revised Child Anxiety and Depression Scale (Chorpita et al., 2000); ADIS-C/P CSR = Anxiety Disorders Interview Schedule for Children and Parents Clinician Severity Rating (Silverman and Albano, 1996); SCQ-F = Social Cognitions Questionnaire – Frequency subscale; SCQ-B = Social Cognitions Questionnaire–Belief subscale; SAQ – Social Attitudes Questionnaire; SBQ = Social Behaviour Questionnaire; PHQ-9 = Patient Health Questionnaire (Kroenke, Spitzer and Williams, 2001); GAD-7 = Generalized Anxiety Disorder Screener (Spitzer, Kroenke, Williams and Löwe, 2006).

Box 1. **Vignettes**

**Patient 1** is a 15 year-old-girl with a 4-year history of social anxiety disorder and depression^1^. She has previously received a course of broad-based CBT for social anxiety. At assessment she struggles to speak or make eye contact, does not smile, often fidgets and wrings her hands. In class she struggles to concentrate, explaining that “my mind gets so caught up in my thoughts I can't focus on what the teacher is saying”. She is worried about her GCSE examinations. She does not participate in class or ask for help when stuck. She pretends to be writing when the teacher passes to avoid being asked a question. She has three friends in school. At home, she does not socialize, fearing she will “ruin the atmosphere because I am so awkward”. She declines invitations from friends. She replies to text messages with brief responses but does not use the telephone. She does not allow herself to be photographed. She wears dark colours so as not to be noticed.

For all adolescents, treatment followed the protocol we have outlined. Each adolescent's key thoughts and safety behaviours are shown in Table 1. Particularly important interventions for Patient 1 included the use of video feedback and of still photographs of her with her friends to update the distorted image of herself as a “stone”, separate from everyone. These provided realistic information about how she actually appeared to others. As well as recovering from social anxiety, Patient 1 achieved all A* to B GCSE examinations, she developed a large group of friends, attended birthday parties and arranged and hosted her own 16^th^ birthday celebration. She wore a bright dress to her school prom and shared photos of this with her friends on Facebook.

**Patient 2** is a 12-year-old girl with a 2-year history of social anxiety disorder with panic attacks and angry outbursts^1^. She has previously received supportive counselling. At assessment she is quiet, frequently says, “I don't know”, and is quick to agree with suggestions. She often looks to her mother to answer questions. She has not attended school for 9 months. She experienced bullying in school, near her home and on social media for several years. At home, she does not use the telephone or social media. She responds to, but does not initiate, text conversations. She will not travel alone, eat or drink in public, or socialize without her mother.

A particularly important intervention for Patient 2 was training in an external focus of attention and learning that “the scariest place is in my head”. As well as recovering from social anxiety, she returned to full-time education, developed a new friendship group, and attended a dance-drama club. Angry outbursts and panic attacks resolved.

**Patient 3** is a 16-year-old young woman with a 4-year history of social anxiety disorder, generalized anxiety disorder and depression^1^. At assessment she is visibly anxious, concerned to give the “right answer” and physically keyed-up. She is polite and engaged. At a previous assessment for autism spectrum disorder, obsessive and rigid “autistic traits” were observed, but her developmental history and current functioning were not consistent with a diagnosis. Although Patient 3 is extremely intelligent and high achieving, school initiated the referral to CAMHS because of a one-year history of non-compliant, challenging behaviour in lessons (e.g. putting her head on the desk, refusing to answer questions) and intermittent school refusal. At home, she does not use the telephone. She responds to, but will not initiate, text conversations. She has a small social network but no best friend and has always been socially reserved.

The attention and safety behaviours experiment was a particularly enlightening intervention for Patient 3. She had firmly believed her safety behaviours were helpful but was surprised to learn that they made her feel more anxious and led her to think she came across less well. In addition to recovering from social anxiety, the young person obtained all A*-A grades at GCSE and completed an interview that led to her being awarded a scholarship. She attended her school prom and organized a holiday with friends.

**Patient 4** is a 17-year-old young man with a 6-year history of severe social anxiety disorder, selective mutism and depression^1^. He has not responded to treatment with Fluoxetine (20mg od) nor to a course of exposure based CBT for social anxiety. He experienced severe bullying in two different schools. At assessment he is extremely anxious, struggles to speak, and makes no eye contact. When anxious he chews his hands, which has caused callouses. He has not contributed in class, spoken to another pupil or socialized with a peer for at least 12 months. He sits in a corner of the classroom away from peers and covers his face with his coat (which he wears at all times). He spends his free time in his room on his computer, playing games with others but not interacting beyond this.

Particularly important interventions for Patient 4 included imagery rescripting of memories of severe bullying and use of still photographs to update his self-images. As well as recovering from social anxiety, Patient 4 completed his A-Levels and was offered a place at university. His hand biting resolved. He started to meet with friends in his free time and speak to his peers in class.

**Patient 5** is a 15-year-old girl with a 4-year history of social anxiety and generalized anxiety disorder and depression^1^. She has received treatment for Anorexia Nervosa – restrictive subtype, and has maintained a healthy weight for one year. She has also previously received a course of CBT for Generalised Anxiety Disorder. At assessment she is anxious and tearful but extremely polite and friendly. In school, she does not participate. She is a bright girl but teachers report she is underachieving and she describes difficulty concentrating. At home, she does not use the telephone and she responds to, but will not initiate, text conversations. She has a close group of friends, but does not instigate any arrangements to meet them and often turns down invitations. Her sleep is poor. Parents report frequent conflict.

Particularly important interventions for Patient 5 included spotting her self-critical voice in social situations and using this as a cue to shift attention outwards. As well as recovering from social anxiety, she took the lead role in a play, attended parties, and started making plans to host a small party herself. The family reported a great improvement in relations at home. For example, she and her father described working together to plan her work experience placement.

^1^All diagnoses referred to reflect the current situation at the pretreatment assessment based on assessment with the ADIS-C/P

### Ethical considerations

All patients were treated as part of a routine clinical service and the project was considered clinical audit. All patients provided written consent for the data to be used for publication.

### Measures

To help guide therapy, participants completed measures of social anxiety, mood and concentration in the classroom, and social anxiety related processes before every session. Some additional measures were completed at pre, mid, and posttreatment and follow-up.

#### Outcome measures

The primary outcome measure was the Liebowitz Social Anxiety Scale: self-report version (LSAS; Baker, Heinrichs, Kim and Hofmann, 2002). The Social Phobia Weekly Summary Scale (SPWSS; Clark et al., 2003) was used as an additional measure of social anxiety. This was designed for use with adults but has been used successfully with youths. Concentration in class was assessed by asking young people to rate their ability to concentrate on class or learning activities using a visual analogue scale ranging from 0 (not at all) to 100 (totally).The Revised Child Anxiety and Depression Scale (RCADS; Chorpita, Yim, Moffitt, Umemoto and Francis, 2000), which has a social anxiety subscale, was completed by parents (used to refer to parents/carers throughout) pre, mid, and posttreatment and at follow-up.

#### Social anxiety process measures

Several unpublished measures (Clark, 2005) covering central processes in cognitive models of social anxiety (Clark and Wells, 1995; Rapee and Heimberg, 1997) were administered and used to help guide therapy. The Social Cognitions Questionnaire (SCQ) is a 22-item scale covering negative automatic thoughts that are commonly reported in social anxiety provoking situations. Two subscales scores are obtained: a mean thought frequency, ranging from 1 (thought never occurs) to 5 (thought always occurs when I am anxious); and a mean belief rating ranging from 0 (I do not believe this thought) to 100 (I am completely convinced this thought is true). The Social Attitudes Questionnaire (SAQ) is a 41-item scale measuring social anxiety related beliefs. Each item is rated from 1 (totally disagree) to 7 (totally agree), and a mean score is obtained. The Social Behaviour Questionnaire (SBQ) is a 29-item scale measuring how often individuals use a range of common safety-seeking behaviours in social situations. The frequency with which each behaviour is used in social situations is rated from 0 (never) to 3 (always), and a mean score is obtained. The SCQ, SBQ and SAQ have been shown to discriminate between high and low socially anxious youth in UK (Hodson et al., 2008) and German (Schreiber, Hoefling, Stangier, Bohn and Steil, 2012) samples. In the present study, the SCQ was completed weekly. The SAQ and SBQ were completed at pre, mid and posttreatment and at follow-up.

#### General mood measures

Depression and general anxiety were assessed fortnightly with the Patient Health Questionnaire-Depression scale (PHQ-9; Kroenke, Spitzer and Williams, 2001) and the GAD-7(Spitzer, Kroenke, Williams and Löwe, 2006) respectively.

#### Service user feedback

The Experience of Service Questionnaire (CHI-ESQ; Attride-Stirling, 2002) was completed once at the end of treatment by parents and young people.

#### Criteria for assessing remission

Remission was assessed in two ways. Jacobson and Truax's (1991) criteria for reliable and clinically significant change were applied to LSAS scores. Pre–post change had to exceed the measurement error of the scale *and* move individuals into the distribution of the non-clinical population (mean ± 2 *SD*). Using Fresco et al.’s (2001) data for a non-clinical population, this equated to a pretreatment to posttreatment fall of at least 12 points on the LSAS and a posttreatment LSAS score of ≤37. In addition, EL reassessed participants on the ADIS-C/P criteria for social anxiety disorder at the end of treatment.

### Therapist and supervisor

As the aim of the case series was to learn how to adapt CT-SAD for use with adolescents, it was essential that the therapy and supervision were conducted by individuals who were fully trained in CT-SAD for adults. All sessions were delivered by EL, an experienced child clinical psychologist who had been trained in CT-SAD with adults during a clinical placement at the Centre for Anxiety Disorders and Trauma at the Maudsley Hospital in London. DC, one of the originators of CT-SAD, provided weekly supervision via Skype.

### Treatment

Therapy was delivered in line with the standard adult protocol (i.e. 14 individual 1.5 hour sessions with follow-up appointments at 1, 2, and 3 months). After each session, EL carefully reviewed the session videotape and discussed with DC what happened in the session along with her plans for the next session. Any apparent relationships between change in social anxiety and procedures used in a preceding session were noted, as were adaptations or refinements to the treatment procedures that appeared to be helpful.

**Figure 1.**
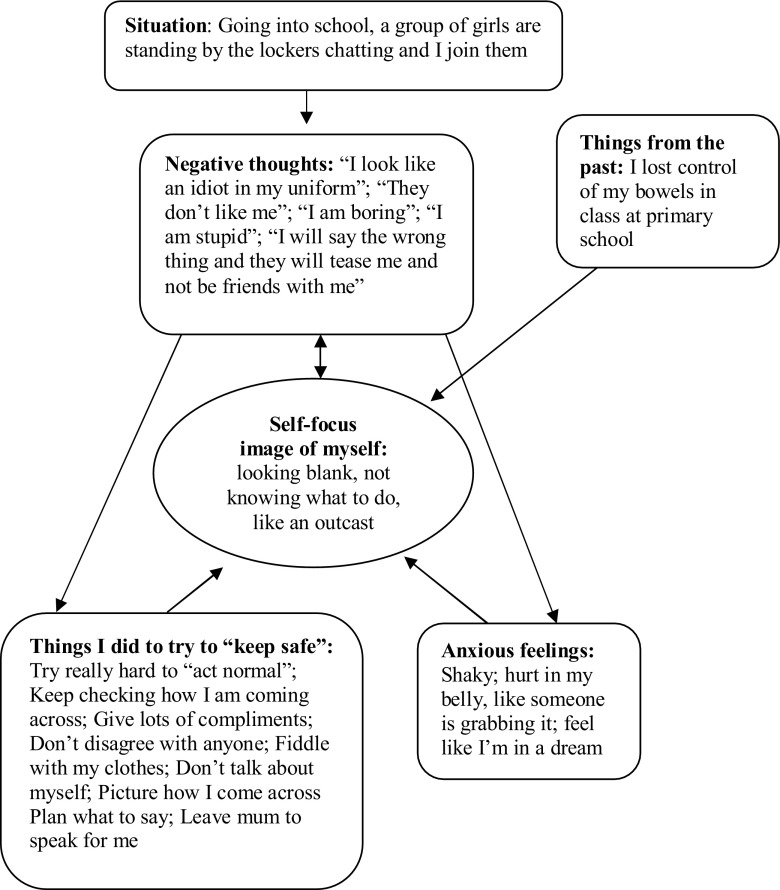
Idiosyncratic formulation for Patient 2

1) An individualized version of Clark and Wells’ (1995) model is collaboratively developed with the young person using their own thoughts, images and safety-behaviours. The model is drawn on a whiteboard as looking at the board, rather than directly facing the therapist, helps the young person to feel less self-conscious in the initial session. Figure 1 depicts Patient 2’s model.
2) Young people undertake an experiential exercise to help them discover the unhelpful consequences of self-focused attention and safety behaviours. In Session 2 they have two conversations with a stranger (due to availability this is usually an adult). In the first conversation, they focus their attention on themselves and think how they are coming across to the other person, while also engaging in their habitual safety behaviours (self-focused, evaluative attention condition). In the second conversation, they are encouraged to focus externally, not to think how they are coming across and instead get involved in the topic of the conversation, without doing their safety behaviours (externally focused, non-evaluative attention condition). Typically, young people discover that their habitual approach to social interactions (self-focused, evaluative) makes them feel more anxious and they think they appeared more anxious and performed less well. The young person is not told in advance what to expect because we prefer them to learn these key points through experience. Homework involves repeating this exercise with a peer.
3) Video (and photograph) feedback is used to update distorted negative images and impressions of the way one appears to others. The first video feedback exercise occurs in Session 3 when young people are asked to specify how they think they came across in each of the conversations in Session 2 and then to compare their predictions with how they actually look on the video. For behavioural experiments outside the office, young people can use their mobile phones to collect a video or pictorial image to compare with their negative self-impressions.
4) Young people receive a session of systematic training in externally focused, non-evaluative attention. This starts by attending to external non-social stimuli (street sounds, music, colours) and progresses to social situations. Young people use the classroom setting to practise externally focusing attention on a daily basis.
5) Behavioural experiments are undertaken in most therapy sessions. We collaboratively devise experiments to test out specific beliefs the young person has about themselves and their social world. The young person is encouraged to drop their safety behaviours and focus their attention externally during the experiment in order to gather new information about their anxious predictions. These aim to help the young person discover that the feared consequence is less likely to occur than they had believed (see Box 2 for an example with Patient 3). Experiments can also involve *intentionally* behaving in an “unacceptable” way and examining the consequences (see Box 2 for an example with Patient 5), in order to “decatastrophize” the young person's fears. This latter form of experiment is usually introduced in the second half of therapy. Peer interactions are typically seen as the most threatening by adolescents, but they are difficult to arrange in clinic. To overcome this problem careful planning of homework tasks is required, involving liaison with parents and teachers.
  Box 2. **Example behavioural experiments**
  **An experiment examining likelihood of a feared consequence occurring with Patient 3**
  *Situation:* Parents’ evening
  *Prediction:* “I will speak at the wrong moment, make a stupid comment, the teacher will look at me disapprovingly and I will embarrass mum and dad” [Belief rating: 95%]
  *Experiment:* I made a comment when it came to mind, without preparation and focused on the teacher's reaction.
  *Outcome:* The teacher listened to what I said and everyone seemed glad that I'd contributed.
  *What I learned:* “I can give my opinions when I want” [Belief rating: 80%]
  *How much I believe my original prediction now:* 0%
  **A “decatastrophizing” experiment with Patient 5**
  *Situation:* In the park with friends
  *Prediction:* “I will not agree with what someone says and they will think I am naïve and a fool, and stop talking to me” [Belief rating: 100%]
  *Experiment:* When someone asked if I had watched a movie I said “no”, then stayed focused on the situation to find out what happened.
  *Outcome:* The person didn't look unimpressed and it started a whole new conversation about really famous movies we've never got round to watching and other people joined in.
  *What I learned:* “Differences between people are interesting; I can be honest about myself” [Belief rating: 75%]
  *How much I believe my original prediction now:* 0%
6) When young people identify previous, socially traumatic experiences related to their current concerns and negative self-images, discrimination training or memory rescripting (see Wild and Clark, 2011) is used.
7) Anticipatory worry and postevent rumination are targeted. The first step involves helping the young person discover that these are unhelpful. Once we have done this we explain that instead we will focus on testing out beliefs *in action*.
8) For those young people with negative beliefs about themselves that are not confined to social performance, a self-esteem component is included. Helpful metaphors include: “treating yourself as a friend, rather than being your own bully”, and “being on your team”.
9) The degree of parental involvement varies depending on the extent to which problematic parental beliefs and behaviours are identified. Components that will be important with the majority of parents include: psychoeducation about social anxiety and cognitive therapy; and learning about a child's relapse prevention plan and their role in supporting this. Where problematic beliefs and behaviours have been identified additional elements include helping parents: recognize their own social attitudes; how these relate to their beliefs about their child; how they impact on their own behaviour and interactions with their child; and then testing out alternative responses. Delivery techniques include: Socratic questioning, modelling and behavioural experiments.
10) Liaison with school is helpful at assessment and throughout treatment. First, in order to obtain information about the young person's social and academic functioning, concentration and attention, and behaviour. Second, to support school through the young person's treatment. This might include psychoeducation about social anxiety and cognitive therapy and how teachers can help at the different stages of treatment e.g. planning school-based behavioural experiments.
11) Bullying and its consequences are addressed in various ways. Current bullying is tackled directly in a robust manner through liaison with school and parents. In addition, young people are helped to disengage from victimizing groups and seek out like-minded peers. For example, Patient 2 was enrolled at a dance-drama club that provided a supportive environment in which to test out her social fears.
12) Finally, a therapy blueprint is developed, drawing together what has been covered in sessions and detailing how to continue to build on progress made.

Several CBT procedures that are commonly used in other treatment programmes are *not* used in CT-SAD. These are: repeated exposure to promote habituation; exposure hierarchies; rating anxiety in feared situations (SUDS); thought records; rehearsal of rationale responses in social situations (self-instruction); social skills training.

## Results

### Clinical outcomes

**Figure 2.**
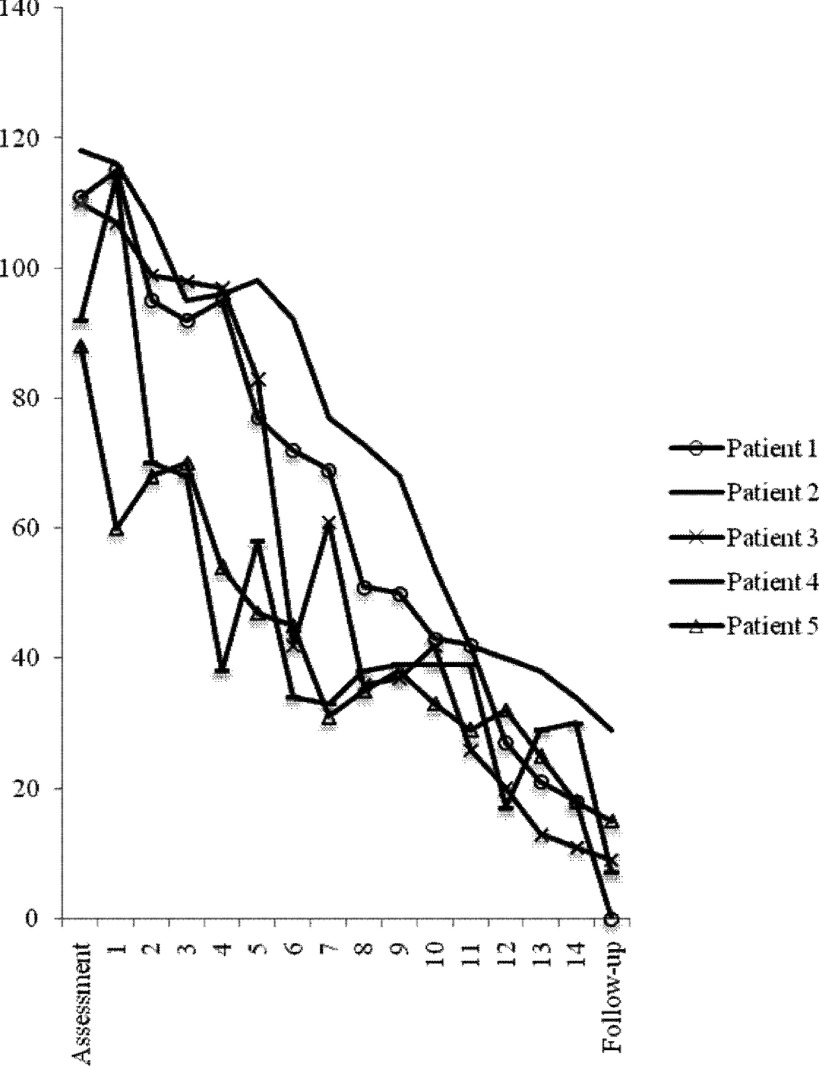
Session by session LSAS score for each patient

By the end of treatment, all patients had lost the diagnosis of social anxiety disorder and ADIS CSR scores were three or below (see Table 2). All met Jacobson and Truax (1991) reliable and clinically significant change criteria. There were large changes in LSAS scores (see Table 2 and Figure 2), with the mean decrease being 79.2% at posttreatment and 88.4% at follow-up. There were large reductions in SPWSS scores and consistent increases in classroom concentration (see Table 2). Parent reported social anxiety symptoms also reduced over time.

### Social anxiety process measures

Scores on the SCQ (frequency and belief subscales), SAQ and SBQ all showed large reductions (see Table 2).

### General mood

Scores on a measure of depression (PHQ-9) and general anxiety (GAD-7) also reduced (see Table 2).

### Satisfaction and functioning

All participants attended all sessions. All CHI-ESQ items were rated as “partly” or “certainly” true, indicating high treatment satisfaction. All participants were attending school full-time by the end of follow-up.

## Discussion

Our development case series provides preliminary evidence that CT-SAD can be adapted for adolescents with excellent results. All five young people initially presented with severe and chronic social anxiety. All had comorbid anxiety disorders and/or depression. By the end of treatment, symptoms of social anxiety, as well as associated anxiety and depression, had reduced to subclinical levels and these gains were maintained at follow-up. These results are particularly impressive when one bears in mind that four of the five young people had already received a standard course of CBT without apparent response. All the young people also showed improved functioning, as evidenced by increased social participation and 100% school attendance at follow-up. Excitingly, we have the first indication that social anxiety treatment may also have a positive impact on classroom concentration, as evidenced by the self-reported improvement across all five patients. Patient 5 explained that: “it is easier to understand things in class now because I can listen to the teachers’ whole explanation, not just patches.” This is important as social anxiety disorder has a particularly deleterious effect on academic functioning (Van Ameringen, Mancini and Farvolden, 2003). Finally, CT-SAD appears to be acceptable to young people and their families. No one missed a session and feedback from parents and young people was consistently positive.

The average change (79%) on the primary outcome measure (LSAS) was greater than observed in our trials of CT-SAD in adults (57% and 63% in Clark et al., 2003, 2006). A similar increase in effect occurred when we adapted our adult cognitive therapy for PTSD for use with children and adolescents (see Smith et al., 2007 vs Ehlers, Clark, Hackmann, McManus and Fennell, 2005). This suggests that CT programmes that tightly focus on the maintenance factors that are prominent in the anxiety-related disorder being treated *may* be particularly effective in young people, a finding that is perhaps somewhat at odds with the tendency to use more broad-based CBT programmes with youth. However, our findings are based on a small number of patients and need to be confirmed in a larger sample. As this was a development series, we spent an unusual amount of time reflecting on the sessions and discussing them in supervision. The results are promising but randomized controlled trials that compare CT-SAD with other approaches to treating social anxiety in adolescents are required to clearly establish the relative efficacy of the treatment approach.

Treatment generally followed the adult protocol, which we found was acceptable to young people. They were able to identify negative social cognitions, a self-focused attentional style, safety behaviours, and distorted self-images. Each of these putative process variables changed with treatment. The self-images often related to earlier socially traumatic events. As in adults, these events appeared to generate a particular (distorted) mental representation that persisted, even though it may no longer be appropriate. The treatment was adapted throughout to ensure it was developmentally sensitive. We also included additional procedures in order to target the following factors: parental cognitions and behaviours; peer victimization and bullying; and low self-esteem.

Unhelpful beliefs were identified and addressed with at least one of the parents of each adolescent. Some parents viewed their child's the social environment as hostile and/or something their child as unable to cope with it, leading to overprotection. Other parents believed that children had to learn to be “centre-stage” and pushed the child too hard, given their anxious concerns. Towards the end of therapy, we found it powerful to invite parents to hear the therapy blueprint their child had developed. We encouraged young people to take the lead: talking through what they had learned about the development and maintenance of social anxiety, how to tackle it, the progress they had made and future goals, and how to manage setbacks.

Unfortunately, four of our five young people were experiencing peer victimization or bullying. Interventions included liaison with schools and parents to address this, and discussions with young people that helped them develop relationships with children who were not bullies, as well as challenging the idea that the bullies are the arbiters of one's worth. Low self-esteem was a focus in treatment for all those young people who had been subjected to peer victimization or bullying. Whilst unconditional assumptions such as “I am weird” are common in social anxiety, when young people experience bullying these beliefs are repeatedly (and sometimes explicitly) confirmed by peers, leading to more general low self-esteem. Additional techniques included noticing the self-critical voice and “being your own cheerleader rather than your own bully”.

In summary, it appears that although CT-SAD was developed as a treatment for adult social anxiety disorder, it shows promise as a treatment for adolescents when suitably adapted for this population. Further research is required to compare its efficacy with other approaches.
